# Impact of Scattering Foil Composition on Electron Energy Distribution in a Clinical Linear Accelerator Modified for FLASH Radiotherapy: A Monte Carlo Study

**DOI:** 10.3390/ma17133355

**Published:** 2024-07-07

**Authors:** James C. L. Chow, Harry E. Ruda

**Affiliations:** 1 Radiation Medicine Program, Princess Margaret Cancer Centre, University Health Network, Toronto, ON M5G 1X6, Canada; 2 Department of Radiation Oncology, University of Toronto, Toronto, ON M5T 1P5, Canada; 3 Centre of Advance Nanotechnology, Faculty of Applied Science and Engineering, University of Toronto, Toronto, ON M5S 3E4, Canada; harry.ruda@utoronto.ca; 4 Department of Materials Science and Engineering, University of Toronto, Toronto, ON M5S 3E4, Canada

**Keywords:** FLASH radiotherapy, FLASH effect, scattering foil, electron beam, Monte Carlo simulation, electron energy distribution, linear accelerator

## Abstract

This study investigates how scattering foil materials and sampling holder placement affect electron energy distribution in electron beams from a modified medical linear accelerator for FLASH radiotherapy. We analyze electron energy spectra at various positions—ionization chamber, mirror, and jaw—to evaluate the impact of Cu, Pb-Cu, Pb, and Ta foils. Our findings show that close proximity to the source intensifies the dependence of electron energy distribution on foil material, enabling precise beam control through material selection. Monte Carlo simulations are effective for designing foils to achieve desired energy distributions. Moving the sampling holder farther from the source reduces foil material influence, promoting more uniform energy spreads, particularly in the 0.5–10 MeV range for 12 MeV electron beams. These insights emphasize the critical role of tailored material selection and sampling holder positioning in optimizing electron energy distribution and fluence intensity for FLASH radiotherapy research, benefiting both experimental design and clinical applications.

## 1. Introduction

External beam radiotherapy (EBRT) is a modern cancer treatment, utilizing high-energy radiation beams, typically X-ray photons, electrons and protons, to target and destroy cancer cells. This modality allows for the precise delivery of radiation to tumors while minimizing exposure to surrounding healthy tissues [1]. Advances in EBRT have led to the development of various techniques and equipment, including medical linear accelerators (linacs), which are capable of generating and modulating these high-energy beams [2]. Linacs have become the primary apparatus to produce photon and electron beams for EBRT due to their versatility and precision, offering a range of energies in megavoltage that can be tailored to the specific requirements of different tumor types and locations. The continuous improvement of these technologies has significantly enhanced the therapeutic ratio, improving patient outcomes by maximizing tumor control and minimizing side effects [3,4].

FLASH radiotherapy represents an important advancement in the field of EBRT. Unlike conventional radiotherapy, which delivers radiation at standard dose rates over longer periods, FLASH radiotherapy administers ultra-high dose rates of radiation within extremely short time frames. This novel approach, delivering the same doses in milliseconds rather than minutes, has garnered significant attention due to its potential [5]. FLASH radiotherapy has demonstrated efficacy in treating a variety of cancers, including brain, lung, breast, gastrointestinal, and skin cancers. It is noted for its ability to preserve normal tissue integrity during treatment while effectively managing tumors, thereby reducing the likelihood of metastasis. The concept of FLASH radiotherapy emerged from preclinical studies demonstrating that normal tissues exhibit a remarkable resistance to radiation-induced damage when exposed to dose rates exceeding 40 Gy/s, a phenomenon termed the FLASH effect [6]. Recent progress in this area has been driven by the development of specialized linacs capable of producing these ultra-high dose rates [7]. Early experimental studies, both in vitro and in vivo, have shown promising results, suggesting that FLASH radiotherapy could revolutionize cancer treatment by reducing the side effects typically associated with conventional radiotherapy [8]. Ongoing research aims to optimize the technical parameters and understand the underlying biological mechanisms, paving the way for clinical trials and broader clinical implementation [9,10].

FLASH radiotherapy’s ultra-high dose rate creates unique responses in cancerous and normal cells. The rapid delivery generates a burst of ionization events, leading to concentrated reactive oxygen species (ROS) production. This overwhelms repair mechanisms in cancer cells, causing significant DNA damage and enhancing tumor control. Normal cells manage oxidative stress and repair sub-lethal damage more effectively due to robust repair systems. FLASH’s dose rate reduces oxygen consumption in normal tissues, sparing them from typical radiobiological damage, offering a therapeutic advantage [11,12].

FLASH radiotherapy offers several advantages over boron neutron capture therapy (BNCT) [13]. One of the primary benefits is its ability to deliver ultra-high doses of radiation in extremely short bursts, which significantly reduces damage to surrounding healthy tissues while effectively targeting tumors. This rapid delivery minimizes side effects and improves patient comfort. Additionally, FLASH radiotherapy has demonstrated the potential to control tumors and limit metastasis without the need for a complex delivery system or specific boron compounds required in BNCT. This simplicity in implementation can lead to broader accessibility and faster treatment times, making FLASH radiotherapy a promising advancement in cancer treatment.

Beam quality and particle energy distribution also influence these effects. High-energy beams penetrate deeper for targeting deeply seated tumors, while lower-energy beams are advantageous for superficial tumors. Higher energy distributions generate more ROS and oxidative stress, amplifying DNA damage. High-LET radiation produces dense ionization clusters, causing complex DNA damage effective against radio-resistant tumor cells [14,15]. Optimizing beam quality and particle energy distribution maximizes the FLASH effect, ensuring tumor control while preserving normal tissue integrity [16,17,18].

Cell and preclinical models are crucial in advancing the understanding and application of FLASH radiotherapy. These models provide essential insights into the biological mechanisms underlying the FLASH effect, enabling researchers to study the differential responses of normal and cancerous tissues to ultra-high dose rates in a controlled environment. Preclinical studies using animal models help to validate these findings in a more complex biological context, providing data on the safety and efficacy of FLASH radiotherapy before clinical implementation [19]. Moreover, modifying existing medical linear accelerators to produce ultra-high dose rate beams is a feasible and critical step towards translating FLASH radiotherapy into clinical practice [20]. This involves adjusting the linac parameters, such as increasing the electron beam current and optimizing the pulse shape, to achieve the required dose rates [21]. Successful modifications have already been demonstrated in several research settings, proving that clinical linacs can be adapted to deliver FLASH doses [22]. These advancements are pivotal in bridging the gap between experimental research and practical, widespread clinical application, ultimately aiming to improve cancer treatment outcomes substantially.

In electron therapy, the scattering foil serves a crucial role in ensuring the uniform distribution of the electron beam across the treatment area [23]. Electrons emitted from the accelerator tend to form a narrow, pencil-like beam, which is not suitable for treating larger or irregularly shaped tumors. The scattering foil, typically made of a thin metal sheet, disperses the electron beam, broadening it to cover the targeted area more evenly. This process, known as scattering, involves the interaction of electrons with the foil material, causing the electrons to spread out and lose some energy. The choice of material for the scattering foil affects the degree of beam dispersion and energy loss, influencing the dose distribution and penetration depth of the electrons, which are critical factors in effective and precise cancer treatment [24]. In FLASH electron radiotherapy using a modified linear accelerator, the material composition of these foils influences the degree of scattering, energy loss, and spectral distribution of the electrons. Different materials, such as high-*Z* (high atomic number) metals like lead (Pb), can cause significant energy degradation and scattering, resulting in a beam with a lower average energy but increased uniformity. Conversely, using lower-*Z* materials like copper (Cu) can preserve higher energy electrons, reducing the extent of scattering and energy spread. By carefully selecting and optimizing the scattering foil material, it is possible to tailor the beam quality to meet the specific requirements of FLASH radiotherapy [21]. This ensures that the ultra-high dose rates are maintained while achieving the desired energy distribution for effective tumor targeting and minimal normal tissue exposure, thereby enhancing the therapeutic efficacy and safety of the treatment.

The aim of this study is to identify changes in the composition of scattering foils affect the electron energy spectrum of ultra-high dose rate beams utilized in FLASH radiotherapy, using Monte Carlo simulations. The investigation addresses a research gap in understanding the role of scattering foil materials in shaping electron energy distributions for FLASH radiotherapy. This study contributes towards enhancing the precision and efficacy of FLASH radiotherapy treatments, ultimately benefiting patient care and clinical practice. By simulating different scattering foil materials, this study seeks to elucidate the resultant changes in energy distribution and beam quality, which are critical for optimizing the therapeutic effectiveness of FLASH. Monte Carlo simulation, renowned for its accuracy in modeling particle interactions and radiation transport, provides a robust framework for predicting how different materials influence the characteristics of the electron beam [25]. The findings from this study will contribute to the development of more effective and tailored FLASH radiotherapy protocols, potentially improving patient outcomes by enhancing tumor control and minimizing normal tissue damage.

## 2. Materials and Methods

### 2.1. Monte Carlo Simulation, Phase Space File of Linac, and Model Verification

This study utilized the Electron Gamma Shower (EGSnrc) system, code version 2023a, developed by the National Research Council Canada [26]. The EGSnrc-based BEAMnrc code was employed to generate the linear accelerator phase space file [27]. For verification of the Monte Carlo model, a water phantom was constructed using a component module placed under the electron applicator to determine the percentage depth dose (PDD) curves, which was then compared with measurements. The PDD was calculated using the EGSnrc-based DOSXYZnrc code [28].

Phase space files for the 12 MeV electron beams were generated using the BEAMnrc code based on a Varian 21 EX linac (Varian Medical Systems, Palo Alto, CA, USA) [27]. The manufacturer provided detailed descriptions of the accelerator, including geometries and materials of various components such as scattering foils, monitoring ionization chamber, shielding, upper and lower jaws, applicators, and scrapers. The mean energy of the electron beams for the 10 × 10 cm^2^ cutout at the water surface and source-to-surface distance (SSD) = 100 cm was estimated using the measured R_50_ (depth on PDD curve of 50% dose beyond R_100_) with the conversion formula ${\bar{E}}_{o}$ = 2.4 × R_50_. The assumed monoenergetic energy of the primary electron was used to calculate the PDD of the reference 10 × 10 cm^2^ field and compare it to the measurement [29]. The primary electron energy was adjusted after each comparison until the PDD difference between the calculation and measurement was within ±2%, and the measured and calculated R_50_ and R_p_ (electron practical range) were within ±1 mm. R_p_ is defined as the depth at which the dose delivered by the electron beam falls to a specific fraction (10%) of the maximum dose. The primary electron energy, *E*_0_, for the 12 MeV beams used in the Monte Carlo simulation was estimated to be 13.42 MeV [30]. In each simulation, the corresponding phase space file contained particles amounting to at least 20% of the number of histories used in DOSXYZnrc. For instance, for the 10 cm circular field, 45 million histories were used in the DOSXYZnrc calculation, requiring the phase space file to contain at least 9 million particles. This ensures the phase space file is recycled no more than five times during DOSXYZnrc calculations, minimizing statistical artifacts. The EGSnrc transport parameters were set to ECUT = 521 keV, PCUT = 10 keV, and ESTEPE = 0.04. Parameter Reduced Electron-Step Transport Algorithm II (PRESTA II) was employed as the electron-step algorithm with user-adjustable parameters set to their default values [31]. The computing time for generating a phase space file was approximately 1–2 h.

**Figure 1:**
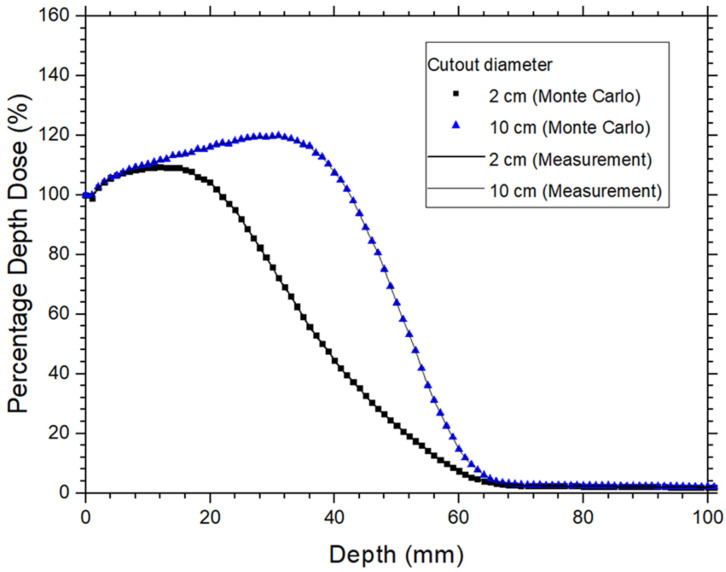
PDD curves measured and Monte Carlo simulated with circular field (cutout) sizes equal to 2 and 10 cm diameter using the 12 MeV electron beams.

The PDD for the verification of the Monte Carlo model was calculated using the DOSXYZnrc code [28]. For circular fields with diameter of 2 cm, the voxel size was set to 0.25 × 0.25 × 0.1 cm^3^ for the 12 MeV beam. For larger circular field of 10 cm in diameter, voxel size was 1 × 1 × 0.1 cm^3^ for the 12 MeV beam. To maintain consistent uncertainty levels across different field sizes, 5 × 10^6^ histories were used for the 2 cm circular field, with the number of histories proportionally increased for larger fields. For example, 25 times more histories were used for the 10 cm circular field compared to the 2 cm field. This approach ensured that the relative dose error (uncertainty as a fraction of dose in the voxel) remained around 1% for all circular fields. Since only the PDD was required for the model verification, a 1D matrix of voxels along the central beam axis was constructed in water for each calculation. The length of each voxel was surrounded by a water medium of sufficient dimensions to provide electronic equilibrium. This method significantly reduced the PDD computing time to approximately 5–20 min [26]. In this study, the ECUT was set at 521 keV in the DOSXYZnrc code. The PDD measurement was conducted using a scanning water tank system (RFA 300, Scanditronix Medical AB with Omni Pro 6 software), typically employed for machine commissioning, and a waterproof high-doped p-type silicon diode (Scanditronix Medical AB, EFD-3G) [32]. Figure 1 presents the PDDs for 2 cm and 10 cm circular fields calculated by Monte Carlo simulation compared with measurements for 12 MeV electron beams. The results show a good agreement between Monte Carlo simulations and measurements within 1%, confirming the accuracy of the Monte Carlo model for the 12 MeV electron beam.

### 2.2. Scattering Foil Material, Position of Sampling Holder, and Calculation of the Electron Energy Spectra

**Figure 2:**
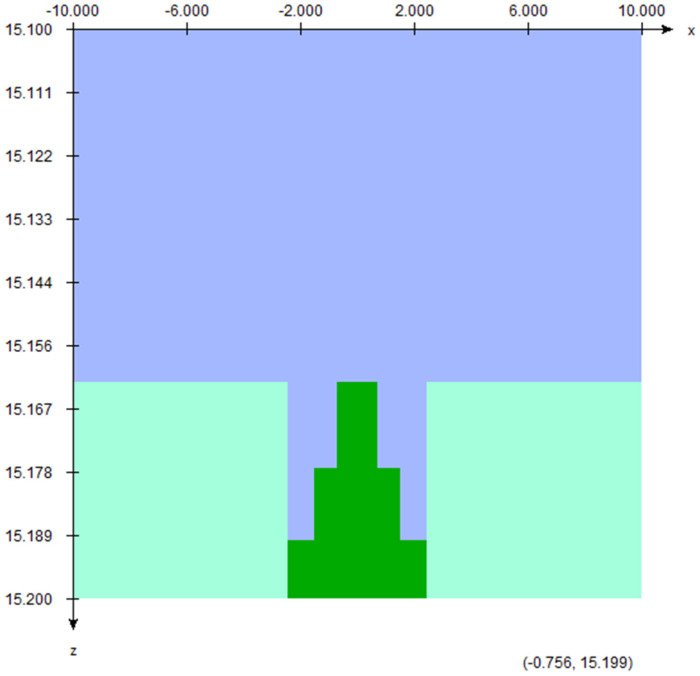
Schematic diagram showing the scattering foil containing three layers. The scattering foil (green) is surrounded by stainless steel (aquamarine) and air (blue).

Figure 2 shows the 2D schematic diagram of scattering foil within the Monte Carlo model, constructed using the BEAMnrc code for a 12 MeV electron beam. The scattering foil shown consists of three layers, each with a thickness of 0.025 mm. In Figure 2, the scattering foil contains three layers (dark green) in the Monte Carlo model. The foil is sur-rounded by stainless steel (aquamarine). For this study, we evaluated both the original configuration—featuring top and middle layers of Pb and a bottom layer of Cu—and foils composed entirely of Pb, Cu, and tantalum (Ta). In the Monte Carlo simulation, the PB521ICRU, CU521ICRU, and TA521ICRU in the EGSnrc-based PEGS4 dataset including various interaction cross-sections were used [27,33]. The density of the Pb, Cu, and Ta layer is equal to 11.35, 8.96, and 16.65 g/cm^3^ in the simulations, respectively. Although each layer’s thickness is standard for the scattering foil setup in the head of the linac, it is important to note that the thickness of foil can influence beam hardening and the energy distribution of the electron beam.

**Figure 3:**
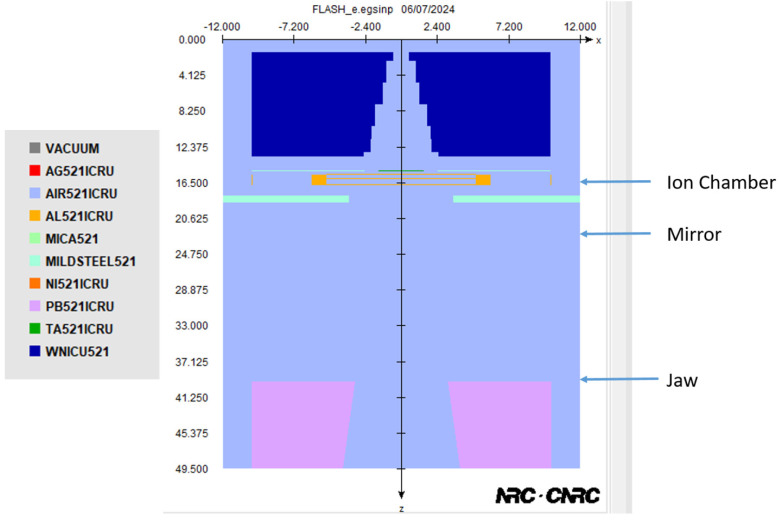
Schematic diagram showing the Monte Carlo model of the modified linac.

The modified linac can position the sampling holder for the cell dish and small animal in three locations within the gantry head [21]: the mirror, ionization chamber, and jaw, as illustrated in Figure 3. The materials for different components are shown in the right of the figure based on the PEGS4 library. The distances from the source to the ionization chamber, mirror, and top of the jaw are 15.6 cm, 19.6 cm, and 27.5 cm, respectively. Figure 3 also depicts the Monte Carlo model, which incorporates materials for various components inside the gantry head based on the PEGS4 database [26].

The electron energy spectra of the 12 MeV electron beam, influenced by various material compositions of the scattering foil, were calculated using phase space files in the Monte Carlo model with BEAMnrc. The phase space files, containing data on multiple crossers, had scoring planes positioned at the ionization chamber, mirror, and jaw locations, as depicted in Figure 3. The BEAMDP code, based on EGSnrc, was employed to analyze the electron energy spectra from the phase space data [34]. Each spectrum was divided into 200 bins, covering an energy range from 0 to 12 MeV. To compare the electron energy distributions at different locations with varying material compositions of the scattering foil, Monte Carlo simulations were conducted, changing the three layers of the scattering foil to Pb, Cu, and Ta.

## 3. Results

**Figure 4:**
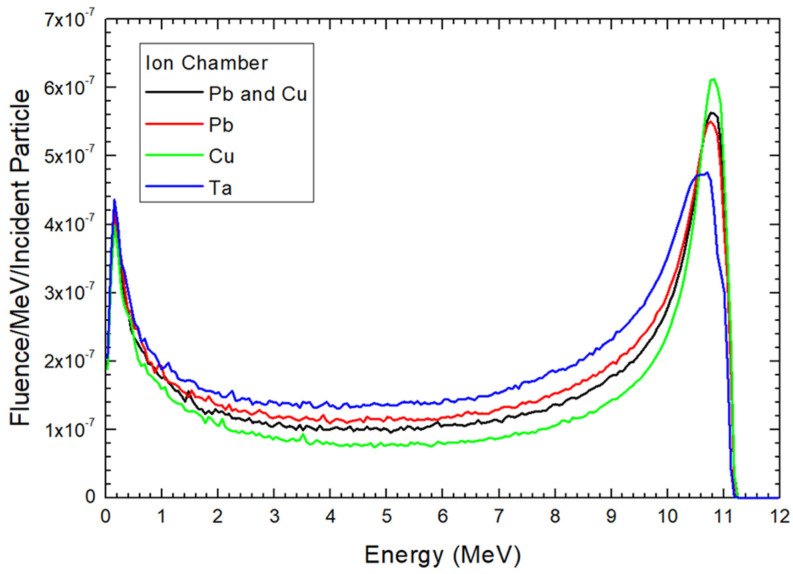
Electron energy distribution at the position of ionization (ion) chamber as shown in Figure 3. The correlation coefficients (4th-order polynomial fitting) for the Pb and Cu, Pb, Cu, and Ta curves are 0.647, 0.664, 0.619, and 0.729, respectively.

**Figure 5:**
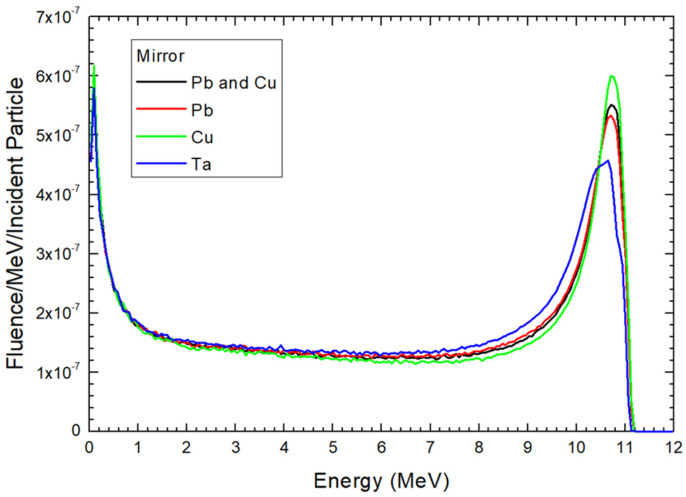
Electron energy distribution at the position of mirror as shown in Figure 3. The correlation coefficients (4th-order polynomial fitting) for the Pb and Cu, Pb, Cu, and Ta curves are 0.576, 0.589, 0.552, and 0.652, respectively.

**Figure 6:**
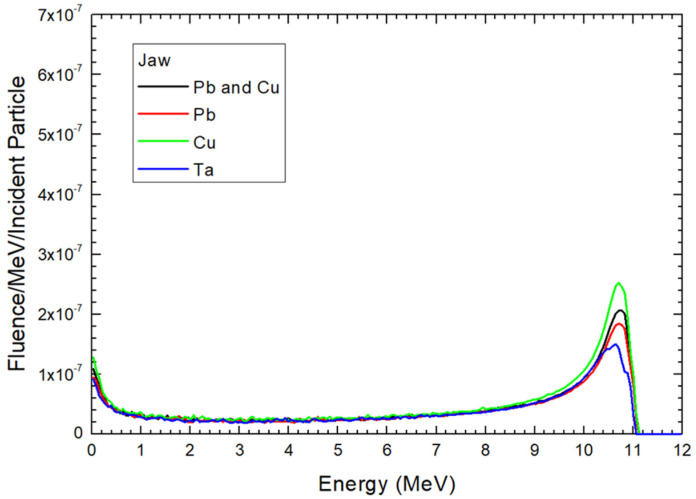
Electron energy distribution at the position of jaw as shown in Figure 3. The correlation coefficients (4th order polynomial fitting) for the Pb and Cu, Pb, Cu, and Ta curves are 0.633, 0.646, 0.618, and 0.692, respectively.

Electron energy distributions for the 12 MeV electron beams at the locations of ionization chamber, mirror, and jaw are shown in Figure 4, Figure 5 and Figure 6, respectively. These locations are the sampling holder positions for the FLASH electron radiotherapy based on the modified medical linac [21]. In each location, the material composition of the scattering foil was changed from Pb and Cu as the original foil composition for the clinical electron beam, to only Pb, Cu, and Ta. In this study, the particle fluence is defined as dN/dA, where dN is the number of electrons entering an imaginary sphere with the cross-sectional area of A. The y-axis of Figure 4, Figure 5 and Figure 6 therefore shows the electron fluence passing through the medium per incident particle per MeV [35]. All electron energy spectra in Figure 4, Figure 5 and Figure 6 were calculated using the same phase space files of specific composition of scattering foil with the same number of particles. The correlation coefficients derived from the 4th order polynomial fitting were calculated using CurveExpert Basic (version 2.2.3) and are provided in the captions of the figures.

## 4. Discussion

### 4.1. Dependences of Electron Energy Distribution on the Scattering Foil Material

Figure 4 illustrates the electron energy distribution for the 12 MeV beam with different scattering foil materials. The beam was located at the ionization chamber position, 15.6 cm from the source. This is the closest distance at which the sampling holder can be placed for an irradiation of ultra-high dose rate electron beam in the modified medical linac. When a pencil beam from the source irradiates the scattering foil, it spreads out to a larger field suitable for covering a tumor. For the original scattering foil used with the 12 MeV clinical electron beam, the first two layers of the foil (see Figure 2) from the source direction are made of Pb, while the largest bottom layer is made of Cu. Heavy-atom materials like Pb are necessary to provide sufficient scattering for the electron beam. However, such heavy-atom layers also cause increased beam attenuation and photon contamination. To address this, a scattering foil composed of both heavy and light-atom layers was developed.

Upon examining the energy peaks in the spectrum for various foil materials as depicted in Figure 4, it becomes evident that a foil composed entirely of Cu exhibits a higher peak compared to a Pb-Cu combination. Conversely, when the foil consists solely of Pb, the peak is lower than both Cu and Pb-Cu compositions. This highlights that Pb, with its higher atomic number, results in higher attenuation of the electron beam, leading to a low peak. However, a higher atomic number does not universally imply a lower peak in the spectrum, as demonstrated by the comparison between Pb and Ta. Despite Pb (*Z* = 82) having a higher atomic number than Ta (*Z* = 73), the energy peak for Ta is lower. This discrepancy arises because the electron attenuation for Ta occurs over a broader energy range (5–12 MeV) compared to Pb (8–12 MeV).

Considering the energy peak in the spectrum for various foil materials as shown in Figure 4, it is found that as the foil is entirely composed of Cu, the peak is higher compared to the Pb-Cu combination. Conversely, when the foil is made solely of Pb, the peak is lower than both the Cu and Pb-Cu compositions. This indicates that the attenuation of the electron beam is highest with Pb, with higher atomic number than Cu, resulting in a low peak. However, a higher atomic number may not absolutely mean a lower peak in the spectrum. This can be seen between the electron energy distribution between Pb and Ta. Although the atomic number of Pb (82) is higher than that of Ta (73), the energy peak of Ta is lower. This is because the attenuation of electron for the Ta layer is distributed in an energy range of 5–12 MeV, which is wider than that of Pb (8–12 MeV).

Although the electron beam energy from the source is nearly monoenergetic, the electron energy distribution downstream of the scattering foil becomes polyenergetic due to electron scattering. As charged particles, electrons are easily influenced by the surrounding medium, which alters their direction and energy. Figure 4, Figure 5 and Figure 6 illustrate that the energy distribution spectrum for different scattering foils exhibits varying fluence intensities, bounded by minimum and maximum energy peaks. In Figure 4, examining the electron energy distribution between 0.5 and 10 MeV reveals that the Ta scattering foil produces the highest fluence intensity among the materials studied. The Pb foil shows higher fluence intensity than the original Pb-Cu foil, while the Cu foil has the lowest fluence intensity. The differences in electron energy spectra due to varying scattering foil materials lead to distinct physio-chemical and biological effects on cells and small animal samples during FLASH radiotherapy [36]. Moreover, the quality of the electron beam can be managed by selecting different foil materials. Monte Carlo simulations can be utilized in the design of scattering foils to achieve a desired electron energy distribution at ultra-high dose rates, which is essential for studying the FLASH effect in cell and preclinical models.

### 4.2. Dependence of Electron Distribution on the Sampling Holder Position

In addition to the ionization chamber location, the sampling holder can be positioned at the mirror and jaw locations in the gantry head for FLASH radiotherapy. These positions are farther from the source compared to the ionization chamber locations. Figure 5 and Figure 6 display the electron energy distribution of the electron beam at the mirror and jaw positions, respectively, when different scattering foils are used.

Comparing the electron energy distribution at the ionization chamber and mirror locations, as shown in Figure 4 and Figure 5 respectively, reveals that the peaks of the spectra are very similar, with fluence intensity following the order of Cu, Pb-Cu, Pb, and Ta. This similarity arises because both locations are close to the source, at distances of 15.6 cm and 19.6 cm, respectively. However, a notable difference between Figure 4 and Figure 5 is observed in the fluence intensity within the 0.5–10 MeV range. Figure 5 shows that the fluence intensity of all scattering foils is very close in this energy range, especially between 0.5 and 8 MeV, compared to Figure 4. This indicates that the greater distance from the source to the sampling holder allows for more electron scattering, resulting in a more uniform energy distribution in the electron beam. Consequently, placing the cell and small animal samples at the ionization chamber location would result in a different electron energy distribution compared to the mirror location, particularly in the mid-energy range.

At the jaw location, 27.5 cm from the source, Figure 6 shows that the fluence intensity of all scattering foils is very close within the 0.5–9 MeV energy range. This greater distance compared to the mirror location (Figure 4) allows more space for electron scattering, resulting in a more even energy distribution. However, the average fluence intensity at the jaw location is significantly lower than at the mirror and ionization chamber locations. In the 0.5–10 MeV range, it is only about 33% and 30% of the fluence intensity at the mirror and ionization chamber locations, respectively. For the maximum peak in the spectrum for the Cu scattering foil, the fluence intensity is just 38% and 26.5% of that at the mirror and ionization chamber locations, respectively. Despite the lower fluence intensity, this is not a significant issue for FLASH radiotherapy, as the dose rate is extremely high, making the additional time to achieve the prescribed dose negligible [10].

### 4.3. Selection of Sampling Holder Location in a Modified Linac for FLASH Electron Radiotherapy

Figure 4, Figure 5 and Figure 6 show that the electron energy distribution of the electron beam depends on the scattering foil material when the sampling holder is placed close to the source, such as at the ionization chamber location. The electron energy distribution is significantly influenced by the foil material, allowing control over the distribution by selecting appropriate materials for the scattering foil. Monte Carlo simulations can assist in designing such foils. However, when the sampling holder is positioned farther away from the source, like at the jaw location, the impact of the scattering foil material on the electron energy distribution, particularly within the 0.5–10 MeV range, becomes negligible. This setup is suitable for cell and preclinical experiments where the focus is not on the FLASH effect related to electron beam quality and energy distribution. Nevertheless, placing the sampling holder at the jaw location results in lower fluence intensity due to the greater distance from the source compared to the ionization chamber location.

## 5. Conclusions

The findings based on Monte Carlo simulation underscore the critical role of scattering foil materials in shaping electron energy distributions for ultra-high dose rate electron beams in modified medical linacs. Results from electron energy distribution reveal distinct energy peaks depending on the composition of the scattering foils. Foils made entirely of Cu exhibit higher energy peaks compared to Pb-Cu combinations, while those composed solely of Pb display lower peaks relative to both Cu and Pb-Cu foils. This trend underscores Pb’s effectiveness in electron beam attenuation, with the intensity of the energy peak influenced by both the atomic number and the range of energy over which electron attenuation occurs, as demonstrated in the comparison between Pb and Ta foils. Additionally, positioning the sampling holder closer to the source in the gantry head leads to more significant variations in electron energy distributions among different foil types. These observations are pivotal for optimizing scattering foils in FLASH radiotherapy, enabling the creation of customized electron energy distributions essential for investigating the FLASH effect in preclinical models and potential clinical settings. The results of this study are anticipated to be valuable for linac engineers, medical physicists, researchers, and clinicians involved in FLASH radiotherapy, especially those investigating the FLASH effect using cell and preclinical models.

## Data Availability

The raw data supporting the conclusions of this article will be made available by the authors on request.
